# International Comparative Study on PISA Mathematics Achievement Test Based on Cognitive Diagnostic Models

**DOI:** 10.3389/fpsyg.2020.02230

**Published:** 2020-09-09

**Authors:** Xiaopeng Wu, Rongxiu Wu, Hua-Hua Chang, Qiping Kong, Yi Zhang

**Affiliations:** ^1^School of Education, Shaanxi Normal University, Xi’an, China; ^2^College of Teacher Education, Faculty of Education, East China Normal University, Shanghai, China; ^3^College of Education, Purdue University, West Lafayette, IN, United States; ^4^College of Education, University of Kentucky, Lexington, KY, United States; ^5^School of Mathematic Science, East China Normal University, Shanghai, China

**Keywords:** PISA, cognitive diagnosis, educational evaluation, international comparison, mathematics education

## Abstract

As one of the most influential international large-scale educational assessments, the Program for International Student Assessment (PISA) provides a valuable platform for the horizontal comparisons and references of international education. The cognitive diagnostic model, a newly generated evaluation theory, can integrate measurement goals into the cognitive process model through cognitive analysis, which provides a better understanding of the mastery of students of fine-grained knowledge points. On the basis of the mathematical measurement framework of PISA 2012, 11 attributes have been formed from three dimensions in this study. Twelve test items with item responses from 24,512 students from 10 countries participated in answering were selected, and the analyses were divided into several steps. First, the relationships between the 11 attributes and the 12 test items were classified to form a Q matrix. Second, the cognitive model of the PISA mathematics test was established. The liner logistic model (LLM) with better model fit was selected as the parameter evaluation model through model comparisons. By analyzing the knowledge states of these countries and the prerequisite relations among the attributes, this study explored the different learning trajectories of students in the content field. The result showed that students from Australia, Canada, the United Kingdom, and Russia shared similar main learning trajectories, while Finland and Japan were consistent with their main learning trajectories. The primary learning trajectories of the United States and China were the same. Furthermore, the learning trajectory for Singapore was the most complicated, as it showed a diverse learning process, whereas the trajectory in the United States and Saudi Arabia was relatively simple. This study concluded the differences of the mastery of students of the 11 cognitive attributes from the three dimensions of content, process, and context across the 10 countries, which provided a reference for further understanding of the PISA test results in other countries and shed some evidence for a deeper understanding of the strengths and weaknesses of mathematics education in various countries.

## Introduction

Initiated by the Organization for Economic Cooperation and Development (OECD) in 1997, the Program for International Student Assessment (PISA) is held every 3 years to assess the fundamental knowledge and critical competencies needed for students approximately 15 years old to participate in society. PISA emphasizes the abilities of students in reasoning from school knowledge and the application of the knowledge to environments outside school (OECD, 2019a). As one of the most influential educational assessment programs globally, PISA has had a large impact on educational practice and reform in many countries by increasing the scopes of tests and strengthening the interpretation of results, thus influencing the decision-making processes for the improvement of national education policies (Breakspear, 2012; OECD, 2013b). For example, the results from PISA 2000 have given rise to a national “PISA shock” in Germany, which has led to massive and rapid educational reforms (Ertl, 2006). Similar educational impacts have also happened in Japan (Takayama, 2008), Denmark (Egelund, 2008), Finland (Dobbins and Martens, 2012), and a number of other European countries (Grek, 2009). The United States, Russia, Japan, and other countries have successively formulated a series of education policies and regulations, forming education quality standards to strengthen the monitoring of the quality of education in the stage of compulsory education. Borrowing from the assessment method of PISA, Singapore has changed the national education assessment model and indicated a new direction for the reform of the national education assessment (Stacey et al., 2015). Mathematics, as one of the core tests in PISA, has also been extensively studied; for instance, educational equity issues have been studied through assessing the opportunities of learning for students (Duru-Bellat and Suchaut, 2005; Luyten, 2017; Hansen and Strietholt, 2018), the gender differences in PISA performance (Steinthorsdottir and Sriraman, 2008; Kyriakides et al., 2014), PISA performance differences in age (Sprietsma, 2010), the relationship between PISA performance and social achievement (Knowles and Evans, 2012), the influence of language on PISA performance (El Masri et al., 2016), the heterogeneity of PISA performance (Wößmann, 2005), etc. However, these studies have focused on either the factors that affect PISA achievements or the impact of PISA achievements on society and education. Few studies have analyzed PISA items, possibly because the PISA items are rarely open to the public. The analyses of the characteristics of mathematics education in different countries through PISA items are of indispensable significance to promote the reform and advancement in mathematics education. To improve the development, mathematics educators, mathematicians, measurement experts, and educational statisticians have been advised to collaborate in research projects to recognize the potential values of concept discussions and secondary analyses that are directly applicable to the existing school systems (Ferrini-Mundy and Schmidt, 2005).

PISA uses item response theory (IRT) in its scaling to overcome the limitations of scoring methods based on number correct or percentage correct. To report the population mean of each subscale, plausible values have been drawn from *a posteriori* distribution by combining the IRT scaling of the test items with a latent regression model using information from the student context questionnaire in a population model (OECD, 2015). Such design is ideal for obtaining accurate rankings for each participating country. However, providing the diagnostic information on the mastery or non-mastery of the examinees of each skill being measured may not be efficient. Under this context, cognitive diagnostic models (CDMs) have risen as advanced psychometric models to support the next-generation assessments aimed at providing fine-grained feedback for students and teachers in the past few decades (Leighton and Gierl, 2007; Templin and Bradshaw, 2014; Chang et al., in press). Researchers have called for additional measurement approaches for reporting and interpreting PISA results (Rutkowski and Rutkowski, 2016). Combining modern statistical methods with cognitive theories, CDMs have been widely utilized in educational and psychological assessment. One of the advantages of using CDMs is their ability to identify the strengths and weaknesses in a set of fine-grained skills (or attributes) when difficulty exists in inferring skill mastery profiles of examinees through traditional methods, such as classical test theory (CTT) and IRT (Choi et al., 2015). Therefore, CDMs have been developed to provide fine-grained information for researchers and educators on the cognitive skills or attributes that are required to solve a particular item, allow applications in various instructional practices, and resolve the limitations that exist in the IRT and CTT models (De La Torre, 2009). By integrating the test objectives into the cognitive process model, CDMs have gained increased attention among the educational and psychological assessments recently (Stout, 2002; Tatsuoka, 2002; Chen and Chen, 2016). Moreover, they can reflect the psychological and cognitive characteristics of the subjects (Templin and Henson, 2010). In the field of mathematics education, diverse cognitive models of mathematics learning and teaching have been developed (Carpenter and Moser, 1982; Greeno, 1991; Rumelhart, 1991; Schneider and Graham, 1992; Zhan et al., 2018) and validated by empirical evidence. It lays a foundation for CDMs that provide the measurement and diagnoses in mathematics educational issues.

The objective of the research is to employ a CDM as an analytic tool to analyze the data set consisting of 10 countries, including China, the United States, Russia, the United Kingdom, Japan, Finland, Singapore, and Australia on the basis of the PISA test contents. The research finding will be based on the mastery levels for the 11 attributes from three aspects, content, process, and context. Through exploring the knowledge states and learning trajectories of the 11 attributes, the study provides new information about mathematics education in the 10 countries regarding the strengths and weaknesses of each the 11 attributes in the study.

## Cognitive Model Construction

Given that PISA tests the fundamental knowledge and key competence necessary for students to participate in the future, the test items are all carried out in specific realistic situations. As far as the mathematics test items are concerned, students need to apply the mathematical knowledge and skills they have learned to solve a practical problem comprehensively. It has a detailed description of the test items. Therefore, an in-depth cognitive diagnostic analysis of the measurement results can be performed according to the existing coding.

### Cognitive Attributes

Attributes play fundamental core roles in cognitive diagnosis measurement. The quality of attributes is directly related to the effectiveness of the cognitive diagnostic evaluation. To some extent, the essence of a cognitive diagnosis is the diagnosis of cognitive attributes. No uniform definition has been given regarding the cognitive attributes in the field of measurement. Attributes are productive rules, project types, program operations, general cognitive tasks (Tatsuoka, 1987), or posited knowledge and thinking skills (Tatsuoka et al., 2004); a description of the procedures, skills, processes, strategies, and knowledge a student must possess to solve a test item (Dogan and Tatsuoka, 2008); or the processing skills and knowledge structure required to complete a certain task (Leighton and Gierl, 2007). The attributes may be of a different nature; they may also be the knowledge, strategies, skills, processes, and methods necessary to complete the task, which is a description of the internal processing of the psychology of students in problem-solving (Cai et al., 2018). Conclusively, the cognitive attribute can be taken as a way of classification to understand the knowledge states of students more precisely on the basis of a certain standard (Wu et al., 2020). According to the definitions of cognitive attributes and the test items provided by the PISA assessment framework, each test item in PISA is defined from three aspects (dimensions), namely, the main subject area involved in the test question, the main mathematical process of problem-solving, and the contexts the test questions are based on (OECD, 2019b). Therefore, the cognitive attributes of PISA test questions can be constructed according to the definition of these three dimensions. We define the term attribute as a mathematical skill or content knowledge that is required to solve a test item. The dimensions, attributes in each dimension, and the corresponding definitions are shown in Table 1.

**TABLE 1:** Dimensions of PISA’s cognitive attributes.

Dimension	No.	Attribute	Definition
Content	N1	Change and relationships	Use mathematical language such as algebraic expressions, equations, functions, inequalities to describe the relationship between quantity and graphic
	N2	Space and shape	Mainly involves the relationship between planes, points, lines, and planes in space, and the virtual rotation of graphics, etc.
	N3	Quantity	Quantity integrates the quantification of the attributes of objects, relationships, situations, and entities in the world, understands the various manifestations of these quantifications, and judges, interprets, and demonstrates the quantity
	N4	Uncertainly and data	Perception of change, probability and opportunity, representation, evaluation, interpretation of uncertainty-centric data
Process	P1	Mathematization	Use mathematical language to describe and explain problems in real life, and convert relevant information into mathematical quantities
	P2	Mathematical operation	Use mathematical concepts, facts, procedures, and reasoning to identify, calculate, reason, and analyze problems
	P3	Mathematical reality	Ability to apply the results of mathematical solutions to real problems and make assessments and inferences on the results
Contexts	C1	Personal	The project’s involvement is based on personal scenarios, mainly focused on the activities of individuals, families or peers
	C2	Occupational	Involving various fields of future work, career scenarios may be related to any level of the workforce, from unskilled jobs to high-level occupational jobs
	C3	Societal	Social issues are concentrated in one’s community, the focus of the problem is the community perspective
	C4	Scientific	Problems in the scientific category involve the application of mathematics in nature, as well as problems and topics related to science and technology

In Table 1, the four attributes in the content dimension include almost all the mathematics content in the stage of compulsory education. This division is relatively clear in maintaining a consistent granularity in the various parts. The three attributes of the mathematical process are the same as the reality, mathematization, and recreation described by the famous mathematician Freudenthal (2012). Mathematical operation is the process of recreation in the field of mathematics, and it is an important method in searching for the essential relationship through a superficial phenomenon. The context attributes include each field that students can encounter in the future, and it is an important carrier for training students to see the world with the “eyes” of mathematics.

### Q-Matrix

Many test items have been included in PISA so far. However, in terms of mathematical tests, only test items publicized in 2012 are available, and no items can be obtained from other years. Even though PISA 2012 has many items, there are only 12 of them jointly tested by the students in the 10 countries we studied. Therefore, this study has selected 12 test items in PISA 2012 for cognitive diagnostic analysis. In PISA, each mathematics item is intended to target all three attributes in one dimension, which can be considered as a latent construct or dimension (OECD, 2014a). The Q-matrix in the cognitive diagnostic assessment we have constructed is a matrix used to connect test items and cognitive attributes, in which 1 represents the corresponding attribute that is considered in the test item, and 0 is the opposite. The Q-matrix has built a bridge between the observable responses of students and their unobservable cognitive states (Tu et al., 2019). According to the mark of the test item in the PISA 2012 manual, the Q-matrix is obtained, as shown in Table 2.

**TABLE 2:** Q-matrix of 12 test items in PISA.

Items	Attributes										
	N1	N2	N3	N4	P1	P2	P3	C1	C2	C3	C4
PM00QF01	0	0	1	0	0	0	1	1	0	0	0
PM903Q03	1	0	0	0	0	1	0	0	1	0	0
PM918Q01	0	0	0	1	0	0	1	0	0	1	0
PM918Q02	0	0	0	1	0	0	1	0	0	1	0
PM918Q05	0	0	0	1	0	1	0	0	0	1	0
PM923Q01	0	0	1	0	0	1	0	0	0	0	1
PM923Q03	0	1	0	0	0	1	0	0	0	0	1
PM923Q04	1	0	0	0	1	0	0	0	0	0	1
PM924Q02	0	0	1	0	1	0	0	1	0	0	0
PM995Q01	0	1	0	0	0	1	0	0	0	0	1
PM995Q02	0	1	0	0	1	0	0	0	0	0	1
PM995Q03	0	0	1	0	1	0	0	0	0	0	1

## Model Selection and Instrument Analysis

### Participants

In this study, the 12 items in PISA 2012 were selected, and the students who completed these 12 items all at the same time were selected as the research objects across the globe. The participants were from the United Kingdom (GBR, 3,811), Finland (FIN, 2,661), and Russia (RUS, 1,666) in Europe; China (CHI, 1,763, including the data selected from Hong Kong, Macau, Shanghai, and other places), Japan (JPN, 1,904) and Singapore (SGP, 1,667) in Asia; the United States (USA, 1,630) and Canada (CAN, 6368) in North America; Australia (AUS, 4,342) in Oceania, and Saudi Arabia in Africa (ALB, 1,402). Given that Brazil, Chile, Colombia, Argentina, and other countries that participated in the PISA 2012 math test in South America did not participate in these 12 tests, no comparable data from South America were available, and no data from Antarctica could be obtained either. The maximum representativeness of the data selection was reached.

### Model Selection

Researchers have developed hundreds of measurement models since the cognitive diagnostic assessment theory was proposed. Measurement models are based on different hypotheses, parameters, mathematical principles, and actual situations. Therefore, the comparison and selection of models have played a vital role in the cognitive diagnosis and evaluation process. A large number of cognitive diagnosis practices have shown that choosing an appropriate cognitive diagnostic model is an important prerequisite for an accurate diagnosis or classification of subjects (Tatsuoka, 1984). To obtain a model with a better fit, this study evaluates the parameters of eight models, namely, DINA (Haertel, 1989; Junker and Sijtsma, 2001; De La Torre, 2009), DINO (Templin and Henson, 2006, 2010), RRUM (Hartz, 2002), ACDM (De La Torre, 2011), LCDM (Henson et al., 2009), LLM (Hagenaars, 1990, 1993; Maris, 1999), G-DINA (De La Torre, 2011), and Mixtures Model (von Davier, 2010). Using the LLM and GDINA packages (version 2.8.0) in software R, 2, 451 datasets for model comparisons are selected from 10 countries through the stratified sampling at a ratio of 10:1 in each country. The comparison results on parameter statistics, such as deviation, Akaike’s information criterion (AIC), and Bayesian information criterion (BIC) are shown in Table 3 below.

**TABLE 3:**
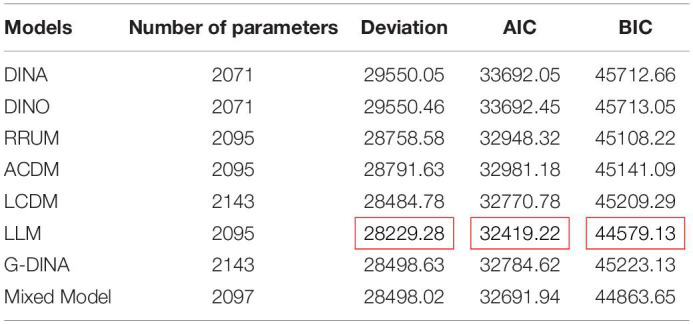
Parameter statistics comparison of different models.

*^∗^Red rectangular box means the smallest index.*

In Table 3, the number of parameters represents the load in the operation of the model, which is closely related to the complexity of the Q-matrix and its attributes. The smaller the number, the smaller the load in the model comparisons. Deviation represents how much an indicator deviates from reality in the model. The smaller the deviation, the greater the degree to which the model fits. In the model comparisons, the AIC and the BIC are mainly used as the reference standards. The AIC is for measuring the goodness of statistical model fit, which is based on the concept of entropy and provides a standard that weighs the complexity of the estimated model and the goodness of the fitted data. The smaller the AIC is, the better the data fits the model. Similarly, the smaller the BIC is, the better the data fits the model (Vrieze, 2012). The results in Table 3 show that the values for Deviation, BIC and AIC of the LLM are the smallest. Therefore, the LLM has a better fit than those in the other models and was preliminarily selected.

### Effectiveness Analysis of the Instrument

#### Reliability

The reliability of the cognitive diagnostic evaluation can be examined from two aspects. One is to treat the test as a common test, and Cronbach’s (α) coefficient is calculated under classic evaluation theory (CTT). The other is to calculate the consistency of the retest of attributes. In our study, we followed Templin and Bradshaw (2013) to estimate the test-retest reliability for our test by simulating repeated testing occasions through repeated draws from an examinee’s posterior distribution. A three-step process is usually used for binary attributes, relying upon the correlation of the mastery statuses between two hypothetical independent administrations of the same test. α = 0.7687 > 0.7, which is an indication of high reliability under CTT theory. The above index of 11 attributes are 0.8941, 0.8372, 0.9124, 0.8541, 0.8193, 0.8512, 0.8135, 0.7942, 0.8135, 0.9721, and 0.9014 accordingly. Data indicators are obtained through the flexCDMs analysis platform (Tu, 2019). The reliability indexes of these attributes are all greater than 0.7. Therefore, they have a high degree of reliability in general.

#### Item Discrimination

Cognitive diagnostic assessment measures the accuracy of cognitive attribute analysis and the quality of test items through item discrimination (Wang et al., 2018). The discrimination degree of the cognitive diagnostic test *d*_*j*_ is defined as

$$d_{j}=P_{j}\left(1\right)-P_{j}\left(0\right),$$

where *P*_j_(1) refers to the probability of mastering all attributes of item *j* when answering the question. *P*_j_(0) refers to the probability of answering the question correctly without mastering all the attributes of item *j*. The smaller *d*_j_ is, the smaller the impact of mastering attributes on the answer is, and the smaller the difference is. In contrast, the difference is greater. A large degree of discrimination is a sign of high-quality test questions. The item discrimination *d*_j_ of the 12 items in this study are in turn equal to 0.902, 0.8497, 0.6901, 0.3174, 0.5758, 0.7457, 0.7716, 0.5213, 0.5912, 0.8078, 0.6142, and 0.5721. All the item discriminations are acceptable except for the fourth item, which is 0.3174. The item discrimination for items 1, 2, 6, 7, and 10 are all greater than 0.7, which has a good discrimination effect.

## Research Analysis and Results

According to the results of the above model selection, the LLM had the best model fit. Therefore, the LLM was used to evaluate the parameters of the research data. The Bayesian expected *a posteriori* estimation (EAP) was used in the process. The Bayesian method attempts to calculate the posterior mean or median rather than a certain extreme value—the mode, the characteristic of which was to use posterior distribution to summarize the data and determine the inference. The posterior estimation is expected to be simple, efficient, and stable, and it is a better choice in the capacity parameter estimation method (Chen and Choi, 2009). The distribution of these 11 attributes from the 24,512 students were assessed initially. Then, the distribution of the attribute in each country was measured. The results for the proportional distribution of the 11 attributes in the 10 countries are in Table 4.

**TABLE 4:** Proportional distribution of 11 attributes in 10 countries.

Country	Content attribute				Process attribute			Context attribute			
	N1	N2	N3	N4	P1	P2	P3	C1	C2	C3	C4
Saudi Arabia	0.246	0.258	0.237	0.388	0.233	0.259	0.248	0.223	0.183	0.381	0.238
Australia	0.273	0.427	0.466	0.699	0.436	0.445	0.526	0.460	0.244	0.701	0.445
Canada	0.411	0.467	0.523	0.742	0.491	0.500	0.578	0.509	0.330	0.741	0.488
China	0.740	0.743	0.749	0.882	0.744	0.753	0.771	0.754	0.693	0.796	0.737
Finland	0.262	0.492	0.586	0.759	0.521	0.554	0.599	0.535	0.204	0.754	0.527
United Kingdom	0.251	0.441	0.491	0.690	0.451	0.450	0.558	0.486	0.215	0.688	0.453
Japan	0.456	0.556	0.568	0.886	0.538	0.558	0.636	0.585	0.431	0.897	0.504
Russia	0.442	0.451	0.441	0.633	0.442	0.454	0.546	0.479	0.366	0.637	0.430
Singapore	0.635	0.651	0.642	0.791	0.635	0.633	0.661	0.651	0.613	0.721	0.636
United States	0.396	0.372	0.425	0.684	0.396	0.407	0.522	0.416	0.297	0.695	0.392

The following discussions are the analyses of the proportional and knowledge states of attribute mastery through the three dimensions of content, process, and context. The proportional distribution of attribute mastery can reflect the differences of attributes in all the countries. Knowledge states can help understand the mastery mode of the attributes of students in different countries and further speculate on the learning trajectories of students.

### Comparative Analysis of Attribute Mastery Probability

#### Content Attribute

**FIGURE 1:**
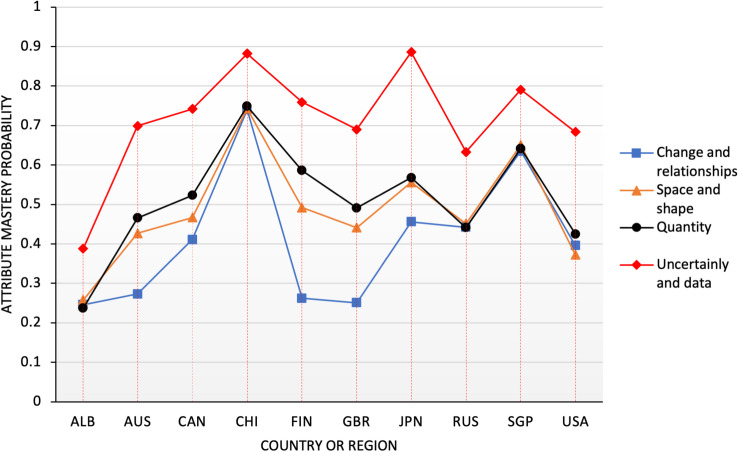
Probability distribution map of content attributes in 10 countries.

The PISA math test involves four content aspects, namely, change and relationships, space and shape, quantity, data and uncertainty, each of which accounts for one quarter of the test (OECD, 2013a). These four overarching ideas ensure the assessment of a sufficient variety and depth of mathematical content and demonstrate how phenomenological categories relate to more traditional strands of mathematical content (OECD, 2010). Almost all content in the junior high school learning has been covered. The probability of mastery of the 10 countries [Saudi Arabia in Africa (ALB), Australia (AUS), Canada (CAN), China (CHI), Finland (FIN), the United Kingdom (GBR), Japan (JPN), Russia (RUS), Singapore (SGP), the United States (USA)] of the four attributes is shown in Figure 1.

As can be seen from the distribution in Figure 1, China performed best in the three attributes of N1 (change and relationships), N2 (space and shape), and N3 (quantity), and it scored much higher than other countries. In the N4 (data and uncertainty) attribute, Japan performed best, and China was second only to Japan. In contrast, Chinese students still had much room for improvement in the study of N4 (data and uncertainty). Moreover, students from China, Singapore, Japan, Finland, and other countries had advantages in grasping each content attribute compared with those in other countries, such as the United States and Saudi Arabia, who showed evident weakness in the content attribute. The result was also consistent with the overall ranking of PISA (OECD, 2014b). In terms of the distribution of the four attributes, all countries performed better in the N4 (data and uncertainty) attribute than in the other three attributes. The United Kingdom, Finland, Saudi Arabia, and Australia had a low level of mastery of the N1 (change and relationships) attribute, less than 30%, and the probability of mastery was less than half of that of China, Singapore, and other countries. The United States performed relatively poorly on the N2 and N3 attributes, especially in the N2 attribute, which was less than half of that of China. On the basis of the above line graph, the differences in content dimensions of the countries can be drawn, which can provide a reference for the countries to formulate curriculum and learning plans. However, change and relationship, as “one of the most fundamental disciplinary aims of the teaching of mathematics may overlap with other content areas in mathematics as it involves ‘functional thinking”’ (OECD, 2013a). Across the globe, algebra and measurement questions were significantly more difficult than number, geometry, and data (OECD, 2010). The students from the United States were strong in some content and quantitative reading skills but weak in others, particularly in geometry (Tatsuoka et al., 2004).

#### Process Attribute

**FIGURE 2:**
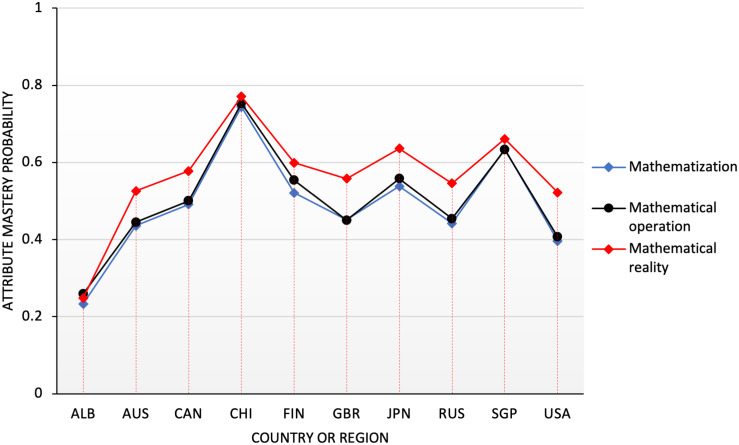
Probability distribution map of process attributes in 10 countries.

The attributes of mathematical processes involved in the PISA math test consist of three aspects, which are the formation of mathematical scenarios; the concepts, facts, processes, and reasoning of applied mathematics; and the interpretation, application, and evaluation of mathematical results, which account for 25, 50, and 25% (OECD, 2013a), respectively. For interpretation convenience, these three processes are abbreviated as mathematization (P1), mathematical operation (P2), and realization (P3). The probability distribution map of the process attributes in 10 countries are shown in Figure 2.

According to Figure 2, the performance of each country in the attribute P3 (realization) was better than others in the process attributes, and no big difference was observed in the performance of P1 (mathematization) and P2 (mathematical operation). China, Japan, and Singapore had a better grasp of the process attributes and a relatively balanced performance. It showed that the students in these countries have reached a very good level in mastering the process attributes. The United States and Saudi Arabia had low performance in the mathematical process, and the development was uneven, especially in the mathematization (P1) attribute. Their performance was much lower, only reaching approximately 25% and approximately one-third of that of China. Meanwhile, the mastery of process attributes in other countries was over 40%. Therefore, a considerable number of students had mastered the process attributes. Overall, students were better in the mastery of the process attributes than the content attributes.

#### Context Attribute

**FIGURE 3:**
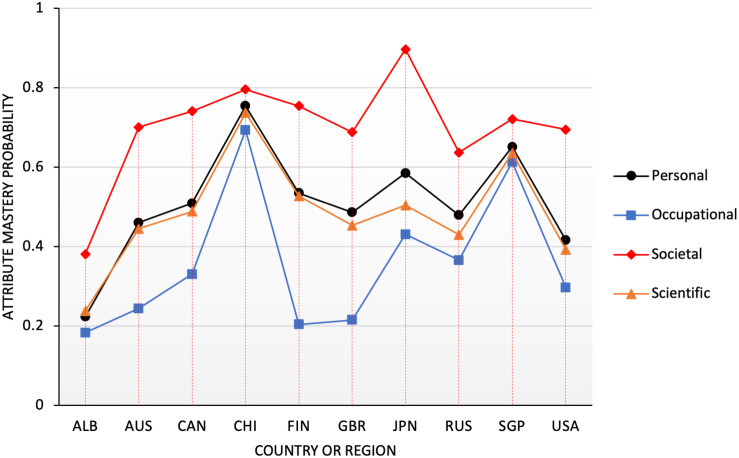
Probability distribution map of 10 countries’ context attributes.

The context questionnaires in the PISA math test involved four parts, namely, the personal (C1), occupational (C2), societal (C3), and scientific contexts (C4). These contexts were necessary for the future student life, and each context accounted for a quarter of the test questions. The context attributes of probability distribution map in 10 countries is shown in Figure 3.

As can be seen from Figure 3, China performed the best in the personal (C1), occupational (C2), scientific contexts (C3) except for the societal contexts (C3) while Japan performed the best in the societal contexts (C3). Singapore had a relatively balanced and good performance for all the four contexts. Additionally, the United Kingdom, the United States, Russia, and Australia had similar performance in the context attribute, and Saudi Arabia performed relatively lower in all the dimensions. In general, the performance of the societal attribute was superior to the other context attributes and the occupational attribute (C2) was worse than the other three attributes. The differences among the personal context and societal context attributes were not large, which reached a relatively certain and balanced level. The occupational attribute (C2) for Saudi Arabic, Finland and the United Kingdom showed an obvious lower performance than the other countries, which accounted for only 20% approximately. At the same time, the probability of mastering the attribute C3 (social situation) was significantly higher than that of the other three attributes.

### Comparative Analysis of Knowledge States

#### Content Attribute

Knowledge states (KS) refer to a set of arrays consisting of 0 or 1. It represents the mastery of the subject of a certain field of knowledge, skills, etc., where 1 indicates that the subject has mastered the corresponding attributes, and 0 indicates that the subject has not mastered the corresponding attributes (Tatsuoka, 2009). For example, (1111) indicates that the subject has mastered all the attributes, and (0010) indicates that the subject has mastered only the third attribute but not the other three. In this study, through the classification analysis of the attributes of each student, the top five knowledge states of content attributes in the 10 countries were counted, and the proportions of the corresponding knowledge states were calculated.

Table 5 shows that seven countries ranked first (1111) in the knowledge states except for Saudi Arabia, Australia, and the United Kingdom, indicating that a large percentage of students had mastered all content attributes. The proportion of knowledge states (0000) in which no attribute was being mastered was also relatively high. Except for China, all the countries ranked in the top two in this attribute, which indicated that a large number of students in most countries did not have any attributes. The knowledge states (0000) in China ranked third, and the proportion only accounted for approximately 10%. The data from almost all the countries supported that the attribute N4 (data and uncertainty) was a prerequisite for the other attributes in the statistical process of knowledge states. The data from Russia further showed that N3 was the premise of N2, and N2 was the premise of N1. Clearly, a linear learning trajectory of N4 → N3 → N2 → N1 was present. The data for Singapore did not show a clear learning trajectory. The so-called learning trajectories were the hierarchical structure of knowledge states, which characterized the relationship among knowledge states with partial order relationships (Duschl et al., 2011). The structure provided a cognitive sequence for learning the content and supported the effective organization of lesson plans and teaching arrangements. A detailed analysis is provided in 4.4.

**TABLE 5:** Top five knowledge states of content attributes in 10 countries.

Country	1st KS		2nd KS		3rd KS		4th KS		5th KS	
	State	Rate	State	Rate	State	Rate	State	Rate	State	Rate
Saudi Arabia	(0000)	0.583	(1111)	0.220	(0001)	0.147	(0101)	0.012	(1100)	0.011
Australia	(0000)	0.302	(1111)	0.263	(0001)	0.230	(0111)	0.161	(0011)	0.033
Canada	(1111)	0.399	(0000)	0.256	(0001)	0.218	(0111)	0.065	(0011)	0.047
China	(1111)	0.739	(0001)	0.136	(0000)	0.108	(0011)	0.007	(0101)	0.006
Finland	(1111)	0.256	(0000)	0.236	(0111)	0.235	(0001)	0.160	(0011)	0.085
United Kingdom	(0000)	0.300	(1111)	0.250	(0001)	0.204	(0111)	0.182	(0011)	0.049
Japan	(1111)	0.452	(0001)	0.311	(0000)	0.113	(0111)	0.108	(1011)	0.012
Russia	(1111)	0.415	(0000)	0.356	(0001)	0.184	(1011)	0.015	(0111)	0.011
Singapore	(1111)	0.620	(0000)	0.196	(0001)	0.141	(0101)	0.014	(0011)	0.010
United States	(1111)	0.354	(0000)	0.317	(0001)	0.248	(1011)	0.036	(0011)	0.025

#### Process Attribute

Table 6 summarized the knowledge states and the corresponding proportion of the top five process attributes in the 10 countries. Except for the United States, Russia, Australia and Saudi Arabia, the knowledge states of the countries (111) ranked first, that is, most students had mastered all process attributes. Similar to the content attribute, the knowledge states (000) were ranked at the top two in all the countries, which showed that some students had not mastered any of the process attributes. Notably, the knowledge states that ranked third in all the countries was (001), which showed that the P3 attribute was particularly important in the learning process. Moreover, this attribute became a prerequisite for learning other process attributes. It was also found that almost all data supported a linear learning trajectory such as P3 → P2 → P1.

**TABLE 6:** Knowledge states of the top five process attributes in 10 countries.

Country	1st KS		2nd KS		3rd KS		4th KS		5th KS	
	State	Rate	State	Rate	State	Rate	State	Rate	State	Rate
Saudi Arabia	(000)	0.710	(111)	0.229	(001)	0.045	(011)	0.014	(010)	0.012
Australia	(000)	0.474	(111)	0.431	(001)	0.075	(011)	0.014	(101)	0.006
Canada	(111)	0.485	(000)	0.418	(001)	0.078	(101)	0.010	(010)	0.004
China	(111)	0.716	(000)	0.194	(001)	0.049	(110)	0.024	(101)	0.007
Finland	(111)	0.523	(000)	0.395	(001)	0.051	(011)	0.027	(010)	0.006
United Kingdom	(111)	0.442	(000)	0.442	(001)	0.100	(101)	0.010	(011)	0.007
Japan	(111)	0.539	(000)	0.362	(001)	0.079	(011)	0.019	(010)	0.002
Russia	(000)	0.453	(111)	0.438	(001)	0.089	(011)	0.016	(101)	0.005
Singapore	(111)	0.629	(000)	0.332	(001)	0.032	(110)	0.004	(100)	0.002
United States	(000)	0.478	(111)	0.386	(001)	0.104	(011)	0.021	(101)	0.010

#### Context Attribute

Table 7 shows the knowledge states of the top five in the context attributes for the 10 countries. China, Canada, Japan and Singapore ranked the first in the knowledge state (1111), which indicated that a considerable number of students had mastered all the attributes in the contexts. The knowledge states (0000) were ranked at the top two in all the countries, which showed that some students had not mastered any of the context attributes. Additionally, attribute (1011) has a higher percentage among most of the countries, which fully explained that the attribute occupational contexts (C2) is a relatively difficult attribute for most students. More importantly, all the countries except for Singapore supported societal contexts (C3) as a prerequisite attribute of other contexts, which provided a cognitive basis for students to solve the mathematics problems. Students tended to approach the problems related to the societal contexts initially and then deal with the problems related to the other contexts.

**TABLE 7:** Knowledge states of the top five context attributes of 10 countries.

Country	1st KS		2nd KS		3rd KS		4th KS		5th KS	
	State	Rate	State	Rate	State	Rate	State	Rate	State	Rate
Saudi Arabia	(0000)	0.555	(0010)	0.143	(1111)	0.090	(1011)	0.086	(0100)	0.024
Australia	(0000)	0.290	(1111)	0.221	(0010)	0.220	(1011)	0.214	(1010)	0.021
Canada	(1111)	0.287	(0000)	0.244	(0010)	0.197	(1011)	0.187	(1010)	0.031
China	(1111)	0.625	(0000)	0.171	(1011)	0.100	(0010)	0.039	(0110)	0.033
Finland	(1011)	0.310	(0000)	0.238	(0010)	0.194	(1111)	0.185	(1010)	0.034
United Kingdom	(0000)	0.293	(1011)	0.243	(0010)	0.209	(1111)	0.199	(1010)	0.029
Japan	(1111)	0.374	(0010)	0.267	(1011)	0.130	(0000)	0.105	(1010)	0.066
Russia	(0000)	0.316	(1111)	0.288	(0010)	0.152	(1011)	0.131	(0100)	0.035
Singapore	(1111)	0.513	(0000)	0.246	(1011)	0.097	(0110)	0.053	(1010)	0.018
United States	(0000)	0.300	(1111)	0.249	(0010)	0.236	(1011)	0.129	(0110)	0.031

### Analysis of Learning Trajectories in the Content Area

**FIGURE 4:**
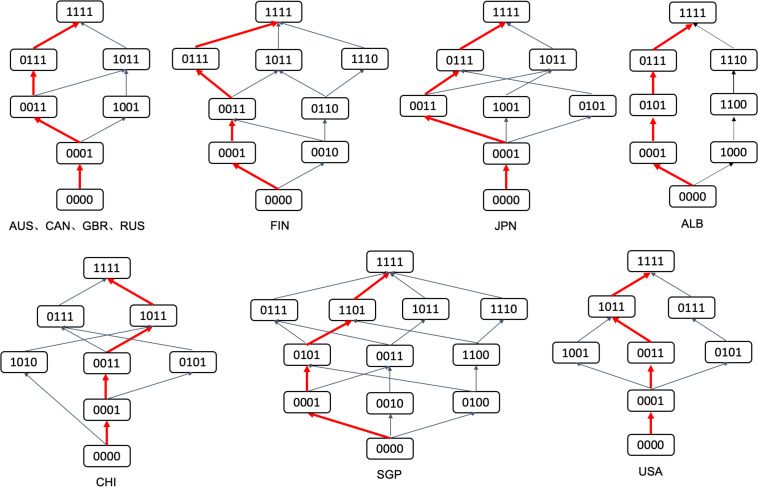
Students’ learning trajectories in 10 countries.

The biggest advantage of the cognitive diagnostic assessment is that it can grasp the cognitive laws of the subjects more deeply. Then, it can design scientific and reasonable learning and remedial programs accordingly. The learning trajectories are related to the development of the cognitive laws of learners and the corresponding arrangement of learning knowledge and skills. It is a learning roadmap that strictly follows the cognitive laws of students. The so-called learning trajectories, that is, the hierarchical structure of knowledge states, characterizes the relationship between knowledge states with partial order relationships (Tatsuoka, 2009). In the process of establishing the learning trajectories, the understanding of students regarding the concepts is assumed to follow the order of easiness to difficulty, that is, students first grasp the basic attributes in the attribute hierarchy, and then grasp higher-order attributes, which are more difficult. Therefore, attributes at lower levels should be easy to grasp, and attributes at higher levels should be difficult to grasp. On the basis of this feature, through the cluster analysis of different knowledge states, the learning trajectories can be drawn on the basis of the inclusion relationship shown in Figure 4. In this diagram, different learning trajectories can be selected for students with different knowledge states.

In the process of construction of the learning trajectories, the knowledge state of each participant is firstly obtained through parameter evaluation, which is the participant’ mastery of each attribute. Then, the participants with the same knowledge state are classified and categorized to establish the trajectory relationship among the knowledge states. The red path is the main trajectory among them, which contains the largest percentage of the participants who own the knowledge states in each level, to some extent, it represents the learning trajectory of a certain group. For instance, in Figure 4, compared with (1010) in the attribute mastering mode, the subjects belonging to the knowledge state (1011) have mastered all the attributes belonging to the knowledge states (1010) and other attributes. Therefore, (1010) ⊂ (1011), which entails an inclusive relationship between the two knowledge states, that is, the trajectory is (1010) → (1011). According to CHI in Figure 4, (0000) ⊂ (0001) ⊂ (0011) ⊂ (1011) ⊂ (1111) exists. Therefore, the learning trajectory in red shown in CHI in Figure 4 is (0000) → (0001) → (0011) → (0111) → (1111). According to Figure 4, Australia, Canada, the United Kingdom, and Russia have the same learning trajectory. The students have three trajectories to master all the attributes from not mastering any attributes in these countries. However, the most important trajectory is shown in red: (0000) → (0001) → (0011) → (0111) → (1111). Most students first obtain N4 (uncertainty and data), then N3 (quantity), then N2 (space and shape), and finally N1 (relationship and change). The learning trajectories of Finland and Japan are more complicated than those of Australia, Canada, the United Kingdom, and Russia. As shown in Figure 4, same as their main learning trajectory, they all follow the trajectory of (0000) → (0001) → (0011) → (0111) → (1111). The learning trajectory in China is also relatively complex, and it has multiple learning trajectories. The main learning trajectory is (0000) → (0001) → (0011) → (1011) → (1111), that is, most students grasp N4 (uncertainty and data) first, then N3 (quantity), and N1 (change and relationships), and finally N2 (space and shape). A difference is observed in the order of obtaining N1 and N2. The learning trajectory in Singapore is the most complicated and has the most diverse learning trajectories. The main learning trajectory is (0000) → (0001) → (0101) → (1101) → (1111), that is, most students grasp N4 (uncertainty and data) first, then N2 (space and shape), then N1 (change and relationships), and finally N3 (quantity). The learning trajectories of the United States and Saudi Arabia were comparatively simple and the main learning trajectories of the United States and China are the same. These trajectories are not only directly related to the cognitive order of students but also influenced by factors, such as national curriculum arrangements and extracurricular tutoring (De Lange, 2007). As can be seen from Figure 4, students in different knowledge states can choose different learning trajectories according to their own characteristics and the learning resources around them, which also reflect the diverse choices of learning. The learning trajectories from the low to the top ends represent different ability levels, reflect the ability relationship among knowledge states, describe the development process of students, and shows the clear development of trajectory and direction for students from low-level learning to high-level learning abilities. Therefore, the learning trajectories not only provide students with personalized and refined diagnostic reports but also provide a basis for the remedial teaching of teachers.

## Discussion

With the advancement of educational globalization, international understanding and international educational references have enabled us to apply the latest achievements to developmental promotion (Wu et al., 2019). PISA, as a product of the development of educational globalization, provides data support for us to understand basic education worldwide and to compare and learn from one another. PISA also reports the motivation, self-confidence, learning strategies, and the environmental background information of students, including the social, economic, cultural, and educational aspects and population distribution related to knowledge and skills (OECD, 2004). The analysis results in this study provided by PISA have surpassed the ranking comparison among the respective fields of countries. It has also offered a unique globalized perspective on how students attain fine-grained attributes and correct the misconception that “correctly answered” items entail that the examinee has attained all the knowledge required to solve the items.

One fact revealed in the PISA 2012 is that all countries that participated have a sizable share of low performers, including those with the highest performance and equity outcomes. On average, 23% of students are low performers in mathematics across all the participating countries; however, the shares of low performers in mathematics vary significantly from country to country (OECD, 2016). Among the 10 countries participating in this study, the proportions of students with low performance in mathematics in the United States, Russia, and Saudi Arabia are higher than average. Additionally, students from different countries have unbalanced performances in various fields of mathematical study. These conclusions are consistent with the above research. The magnitude of the cognitive ability differences between countries is large, and a likely reason for the difference is the Flynn effect, which massively raised the average IQ in economically advanced countries in the 20th century (Meisenberg and Woodley, 2013). Other studies have suggested that the cognitive disparities between advanced industrial societies and less developed countries have been diminishing (Weede and Kämpf, 2002; Meisenberg and Lynn, 2012). Given the positive correlation between IQ (or IQ growth) and economic growth observed, this trend is probably related to a reduction in the degree of economic inequality among countries (Meisenberg and Woodley, 2013).

In terms of the student performances, these 10 countries have large differences in the attributes. However, the conclusion can be quite similar if the students are examined from the aspect of mathematical literacy or creativity. The creativity of students in mathematics is positively related to their achievement in mathematics at the student level within schools. However, the relationship is the opposite among countries (Sebastian and Huang, 2016). Some researchers and educators have realized that academic performance measured through standardized tests narrowly focuses on a few subjects that emphasize identifying correct answers and avoiding mistakes, which ultimately discourages student creativity and critical thinking (Zhao, 2012; Chomsky and Robichaud, 2014; Darling-Hammond and Turnipseed, 2015). On the basis of the findings, consistent top performers in PISA tests, such as Shanghai, Singapore, Korea, and Japan, have started revising their curriculum to increase their emphasis on creative thinking skills (Shaheen, 2010; Kim, 2011). From the aspect of mathematical literacy, Jablonka (2003) discussed the fundamental nature of mathematical literacy. The contexts may be familiar to some students but not to others. Any attempt to use a single instrument to assess mathematical literacy beyond the most local context appears to be self-defeating. Cultural differences exist among countries, and the invariance of the test items also need to be tested accordingly.

In the analysis of learning trajectories, there are two reasons that we only presented the results of content attributes but not the results of the other two attributes. First, in the current research of mathematics education, the learning trajectories are mostly aimed at the its content but not the context or process. Whether there are any learning paths in the process or context attributes, or whether it meets the assumptions of the learning paths, the conclusions are still to be uncovered (Clements and Sarama, 2004; Daro et al., 2011; Wilson et al., 2013). Second, through data analysis, it is found that there are no rules in the process or context attribute, therefore there is no further analysis of the learning trajectories of these two attributes (Confrey et al., 2014). However, the learning trajectories in educational practice is the concept about change longitudinally–how to trace a student’s mastery of the attributes change over time with increasing instruction. The test items in PISA that we studied is cross-sectional due to unavailability of data in the other years. Simply finding relatively large numbers of students in various knowledge states does not imply that individual students move through those states in any specific order. Even if the paths identified reflect reality, there is “correlation does not imply causation” argument to be made; an association between country and these patterns does not imply that specific educational practices lead to those differences. Finally, assuming that the paths are indeed correct, because the data are a single point in time, the silly assertion that these are forgetting paths (i. e., students move from understanding to ignorance) is equally consistent with the data observed. None of this says the learning paths identified are wrong. But any findings from these cross-sectional data are speculative and open to alternative interpretation. They require additional evidence from other sources in order to be evaluated, for instance, studying longitudinally could be an alternative better examination option (Zhan et al., 2019; Zhan, 2020).

Although this study has conducted a more in-depth analysis of the PISA items and results by using the newly emerging measurement method, many areas still need improvement. The study has provided detailed information on 11 attributes in 10 countries in terms of three dimensions, namely, content, process, and context. The division of attributes depends on the coding of existing test questions in PISA without deeper mining in the fields. Later research can divide the attributes in a more detailed way and compare them latently to obtain the advantages and disadvantages of different countries in finer granularity. Additionally, we suggest that questionnaires be sent out to mine the reasons for the difference further. We also need to admit that measuring the change in student achievement at the country level is more robust than measuring student achievement in any single wave of assessment. More methodological and educational research is required to understand the longitudinal trends at the country level (Klieme, 2016). In the end, analyzing the learning situation of students should focus not only on their test scores but also on their external environment, such as the family and school environments. An analysis of the relationship of test scores to variables external to the test can provide another important source of validity evidence (American Educational Research Association, and National Council on Measurement in Education, 2014). Multilevel hierarchical analysis is an important methodology to be taken into consideration in future research.

## Data Availability Statement

The raw data supporting the conclusions of this article will be made available by the authors, without undue reservation.

## Ethics Statement

Ethical review and approval was not required for the study on human participants in accordance with the local legislation and institutional requirements. Written informed consent from the participants’ legal guardian/next of kin was not required to participate in this study in accordance with the national legislation and the institutional requirements.

## Author Contributions

XW designed the study and wrote this manuscript. RW contributed to the manuscript writing and the continued revision provided by the reviewers. H-HC reviewed the manuscript and revised it. QK reviewed the manuscript and provided comments. YZ collected the data and wrote this manuscript. All authors contributed to the article and approved the submitted version.

## Conflict of Interest

The authors declare that the research was conducted in the absence of any commercial or financial relationships that could be construed as a potential conflict of interest.
